# How do disease control measures impact spatial predictions of schistosomiasis and hookworm? The example of predicting school-based prevalence before and after preventive chemotherapy in Ghana

**DOI:** 10.1371/journal.pntd.0011424

**Published:** 2023-06-16

**Authors:** Alexandra V. Kulinkina, Andrea Farnham, Nana-Kwadwo Biritwum, Jürg Utzinger, Yvonne Walz

**Affiliations:** 1 Tufts University – Friedman School of Nutrition Science and Policy, Boston, Massachusetts, United States of America; 2 Swiss Tropical and Public Health Institute, Allschwil, Switzerland; 3 University of Basel, Basel, Switzerland; 4 Ghana Health Service, Accra, Ghana; 5 United Nations University – Institute for Environment and Human Security, Bonn, Germany; University of Cambridge, UNITED KINGDOM

## Abstract

**Background:**

Schistosomiasis and soil-transmitted helminth infections are among the neglected tropical diseases (NTDs) affecting primarily marginalized communities in low- and middle-income countries. Surveillance data for NTDs are typically sparse, and hence, geospatial predictive modeling based on remotely sensed (RS) environmental data is widely used to characterize disease transmission and treatment needs. However, as large-scale preventive chemotherapy has become a widespread practice, resulting in reduced prevalence and intensity of infection, the validity and relevance of these models should be re-assessed.

**Methodology:**

We employed two nationally representative school-based prevalence surveys of *Schistosoma haematobium* and hookworm infections from Ghana conducted before (2008) and after (2015) the introduction of large-scale preventive chemotherapy. We derived environmental variables from fine-resolution RS data (Landsat 8) and examined a variable distance radius (1–5 km) for aggregating these variables around point-prevalence locations in a non-parametric random forest modeling approach. We used partial dependence and individual conditional expectation plots to improve interpretability of results.

**Principal findings:**

The average school-level *S*. *haematobium* prevalence decreased from 23.8% to 3.6% and that of hookworm from 8.6% to 3.1% between 2008 and 2015. However, hotspots of high-prevalence locations persisted for both infections. The models with environmental data extracted from a buffer radius of 2–3 km around the school location where prevalence was measured had the best performance. Model performance (according to the R^2^ value) was already low and declined further from approximately 0.4 in 2008 to 0.1 in 2015 for *S*. *haematobium* and from approximately 0.3 to 0.2 for hookworm. According to the 2008 models, land surface temperature (LST), modified normalized difference water index, elevation, slope, and streams variables were associated with *S*. *haematobium* prevalence. LST, slope, and improved water coverage were associated with hookworm prevalence. Associations with the environment in 2015 could not be evaluated due to low model performance.

**Conclusions/significance:**

Our study showed that in the era of preventive chemotherapy, associations between *S*. *haematobium* and hookworm infections and the environment weakened, and thus predictive power of environmental models declined. In light of these observations, it is timely to develop new cost-effective passive surveillance methods for NTDs as an alternative to costly surveys, and to focus on persisting hotspots of infection with additional interventions to reduce reinfection. We further question the broad application of RS-based modeling for environmental diseases for which large-scale pharmaceutical interventions are in place.

## Author summary

Parasitic worm infections, such as schistosomiasis and soil-transmitted helminthiasis, belong to a group of diseases collectively known as neglected tropical diseases that affect primarily marginalized communities. Transmission of both parasitic worms is affected by behavioral, environmental, and socioeconomic factors. In the absence of reliable surveillance data, sophisticated geostatistical models that rely on environmental predictors are commonly used to predict transmission and treatment needs. We used two nationally representative surveys conducted in Ghana and employed a relatively simple and interpretable modeling approach to predict the risk of *Schistosoma haematobium* and hookworm infections among school-age children before (2008) and after (2015) deworming became a widespread practice. We found that as the proportion of infected children declined, model performance decreased, but hotspots of high-prevalence locations persisted. This suggests that the models may still be useful in spatial targeting of interventions, but the broad application of the approach should be re-evaluated.

## Introduction

Schistosomiasis and soil-transmitted helminth infections are among the neglected tropical diseases (NTDs), affecting primarily the rural poor living in sub-Saharan Africa, Asia, Latin America, and the Caribbean [1]. In Ghana, in the absence of large-scale interventions, the prevalence of schistosomiasis among school-age children was estimated at 23.3% [2] and that of soil-transmitted helminth infections at 19.8% [3]. In regard to schistosomiasis, the predominant species is *Schistosoma haematobium* (22.3%), whereas hookworm (14.4%) is the most frequent soil-transmitted helminth species. The transmission of both diseases is governed by social-ecological contexts, with water, sanitation, and hygiene (WASH) infrastructure and human behaviors playing important roles [4–6].

Due to non-specific symptoms, low treatment seeking behavior, and poor diagnostic capacity in rural communities, routine health management information systems (HMIS) are ill-equipped to detect and report representative case numbers of these helminth infections [7,8]. Transmission and treatment needs at large spatial extents (national, regional, or continental) are characterized primarily through modeling, using periodic school-based or community-based prevalence surveys, coupled with remotely sensed (RS) environmental predictors [2,3,9]. Several improvements have recently been suggested to common modeling methods applied to smaller sub-national spatial extents. These include utilizing fine resolution RS data (e.g., Landsat 8), employing a larger number of relevant environmental indicators derived from the spectral bands (e.g., modified normalized difference water index [MNDWI]), and using a variable distance radius to extract and aggregate environmental indicator variables around point-prevalence locations [10,11].

As large-scale preventive chemotherapy has become a widespread practice that reduced the prevalence and intensity of helminth infections [3,9], the validity and relevance of these models should be re-assessed. In Ghana, school-based preventive chemotherapy with praziquantel against schistosomiasis and albendazole against soil-transmitted helminthiasis was conducted in 2010, 2011, 2012, 2014, and 2015, with coverage ranging between approximately 70% and 85%. In the present study, we examine the limits of the aforementioned environmental modeling approach previously used only in sub-national studies, focusing on the national extent of Ghana. We employed two nationally representative school-based prevalence surveys conducted before (2008) and after (2015) the launch of large-scale preventive chemotherapy, in order to compare model performance before and after a national level intervention. The modeling approach is intentionally relatively simple and easily interpretable, so that it might be used by Ministries of Health to guide planning and monitoring of NTD control measures.

## Methods

### Ethics statement

The study involved secondary data analysis, and hence did not require specific ethical approval. Aggregated school-level *S*. *haematobium* and hookworm prevalence data were provided by Ghana Health Service (GHS). Predictor variables used in the models were obtained from publicly available data sources.

### Study area

The study was conducted in Ghana, a coastal West African nation bordering Togo in the East, Côte d’Ivoire in the West and Burkina Faso in the North. At the time of the study (up to 2015), the country was sub-divided into 10 administrative regions and 216 districts (S1 Fig). Ghana has a variable climate which tends to be hotter and drier in the North and more humid with higher precipitation in the South (S2 Fig). In 2015, approximately 35% of the economically active population were engaged in agriculture [12]. In recent years, Ghana has experienced environmental degradation from deforestation and mining activities [13]. Climate change has resulted in rising temperatures, declining rainfall totals, and periodic flooding [14]. These changes are expected to influence agriculture and food security and environmental disease transmission dynamics, including that of NTDs.

### Data sources

The primary outcome variables were prevalence of infection by *S*. *haematobium* and hookworm among school-age children. The primary predictor variables were land surface temperature (LST), normalized difference vegetation index (NDVI), MNDWI, topographic variables (obtained from satellite RS sources), and WASH variables (obtained from the Demographic and Health Survey [DHS]). Data processing and analysis steps are described below and outlined in S3 Fig.

#### Prevalence data

Schistosomiasis and hookworm prevalence data were obtained from GHS. The national school-based surveys were conducted in 2008 [15] and 2015 in the context of an evaluation study of the impact of the annual national deworming campaign. The baseline survey was conducted between March and May 2008 and included 118 schools. The evaluation survey was conducted between October and November 2015 and included 158 schools. After verification and cleaning (e.g., removing schools for which global positioning system [GPS] coordinates were not accurate), a total of 116 schools remained in the 2008 dataset and 140 in the 2015 dataset. The 2008 and 2015 nationally-representative samples of schools were derived independently.

An average of 50 primary and junior high school students per school (aged mainly between 5 and 14 years) were included in each study. A single urine sample from each child was examined for the presence of *S*. *haematobium* eggs. A single stool sample from each child was subjected to the Kato-Katz technique and tested for the presence of *S*. *mansoni*, and any of the three soil-transmitted helminth (i.e., *Ascaris lumbricoides*, hookworm, and *Trichuris trichiura*) eggs. Individual data for *S*. *haematobium* and hookworm (the other helminth infections were rare) were categorized for presence or absence of parasite eggs and aggregated into school-based percent prevalence values for analysis.

#### RS environmental data

Landsat 8 data were obtained from USGS Earth Explorer (http://earthexplorer.usgs.gov/) as level-2 data products, which had been corrected to remove the effects of the atmosphere on the reflectance values. These products contain spectral bands (#1–#9) to detect surface reflectance values at different wavelengths ranging from 0.44 to 2.29 μm with spatial resolution of 30 m from the Operational Land Imager (OLI), and thermal bands (#10 and #11) covering wavelengths between 10.6 and 12.51 μm with spatial resolution of 100 m from the Thermal InfraRed Sensor (TIRS) [16]. All available images that encompassed the study area (a total of 17 tiles) from October through December 2015 were screened for quality. One image per tile (path and row combination), with acquisition dates ranging between November 20 and December 29, 2015 that was least affected by clouds (<10% of the pixels), was downloaded (S4 Fig). As Landsat 8 images were not available for 2008, we used 2015 images for both surveys, based on the observation that rainfall and vegetation patterns did not change significantly during this time period, visually validated using publicly available climate data [17] (S2 Fig).

ASTER Global Digital Elevation Model (GDEM v2) data were obtained from USGS Earth Explorer (https://earthexplorer.usgs.gov/) with a spatial resolution of 30 m. A moving window (3x3) majority filter was applied to the elevation data to eliminate image artefacts [18,19] using the Spatial Analyst extension in ArcGIS version 10.4.1.

Settlement data were obtained from the German Aerospace Center (http://www.dlr.de) as a Global Urban Footprint (GUF) product. GUF is a binary raster data product of populated and unpopulated pixels produced from 2011–2012 TerraSAR-X and TanDEM-X radar images [20]. GUF was chosen as a source of settlement data due to its 0.4 arcsec geometric resolution, or 12 m spatial resolution, which most closely matched the resolution of the other spatial data used in the analysis.

#### WASH data

Two geospatial interpolated surface layers with a spatial resolution of 5 km were obtained from the 2014 DHS; namely (i) population living in a household using an improved water source (%) and (ii) population living in a household with no toilet facility (%) [21].

#### Population density data

High resolution population density data were obtained from the Data for Good project as a raster image, created by a machine learning algorithm which identifies buildings from satellite images, overlayed with population data from the latest national census (2010), projected to 2020 using the intracensal population growth rate of 2% [22]. This granular dataset with a 30 m spatial resolution represents the total number of individuals living in each 30-m grid cell.

### Data processing

In the Landsat images, pixels affected by clouds or cloud shadows were masked using the quality assurance band. Spectral bands were used to compute NDVI and MNDWI. These vegetation and water indices were chosen out of several that are available due to their better performance in prior analyses in Ghana [23]. Thermal bands were used to derive LST. Data were processed in R software, version 3.6.1.

Elevation data were used to derive stream order and slope. Topographic drainage lines were delineated from the GDEM based on the potential flow direction from higher to lower elevation and accumulation of surface runoff according to topographic conditions using Arc Hydro Tools in ArcGIS, version 10.4.1. The resulting stream network was ordered according to Strahler [24]. Slope of the terrain was derived from the DEM as a proxy indicator for potential flow velocity of surface runoff with inclination calculated in degrees.

The population density data were projected and resampled to match the spatial extent and resolution of all other analysis variables. Because the analysis concerned school-age children, we multiplied each pixel value by 0.24, representing the approximate proportion of children aged between 5 and 14 years among the total population in 2020 [25].

### Variable extraction and aggregation

Aggregated school-based point-prevalence (% positive samples) of *S*. *haematobium* and hookworm were used as the outcome variables. A total of seven environmental predictor variables and two WASH variables were resampled to a matching 30 m spatial resolution for analysis (Table 1). While the predictor variables were represented by continuous raster data, prevalence was represented by point data. Hence, extraction and aggregation of the raster data were necessary. A variable buffer radius (1–5 km) around each point-prevalence location was used by extracting the aggregated (i.e., mean, median, sum, and maximum) pixel value from the buffer area to be matched to each prevalence measure. In addition to five buffer distances, two methods of variable extraction were used; namely (i) no mask (i.e., all pixels within the buffer radius were extracted) and (ii) unpopulated mask (i.e., only unpopulated pixels as defined by the GUF layer were included) [11,23].

**Table 1:** Summary of analysis variables.

*Source*	*Variable name*	*Variable type*	*Resolution*	*Aggregation*	*Value range* *
OLI	NDVI	Continuous	30 m	Median	0.16 to 0.80
OLI	MNDWI	Continuous	30 m	Median	-0.56 to 0.05
TIRS	LST (°C)	Continuous	100 m	Median	21.5 to 38.2
DEM	Elevation (m)	Continuous	30 m	Median	12.0 to 537
DEM	Slope (°)	Continuous	30 m	Median	2.47 to 13.2
DEM	Streams	Binary	30 m	Sum	115 to 3,656
DEM	Stream order	Ordinal	30 m	Maximum	1 to 8
DHS	Access to improved water (%)	Continuous	5 km	Median	29 to 99
DHS	Lack of sanitation facility (%)	Continuous	5 km	Median	0.7 to 98

* Range represents minimum and maximum values present in the dataset (within the largest buffer radius of 5 km in the unmasked dataset).

### Statistical analysis

Exploratory analyses included variable summaries and correlations, followed by random forest models. The random forest approach was chosen because it can deal with continuous outcome data, multicollinear predictor variables, and low numbers of training samples, and hence, it is the recommended machine learning method for generating predictions [26]. It has been successfully applied in similar studies [10,11,23].

We conducted 10 random forest models to determine which of the five buffer distances and two masks present the best method of variable extraction. Each model was applied to the *S*. *haematobium* and hookworm outcomes for 2008 and 2015 study years, resulting in 40 models. Explanatory power was compared using the R^2^ value [27]. Relative importance of predictor variables was assessed using the increasing node purity (‘IncNodePurity’) metric [28,29]. We interpreted the effect of each predictor across the range of its values using partial dependence (PD) and individual conditional expectation (ICE) plots [30].

We applied all models back to the raster stack of predictor variables to derive continuous predicted *S*. *haematobium* and hookworm prevalence surfaces. Although predicted values were available for all pixels, the same masks used to extract the explanatory variables were applied to the respective predicted prevalence surfaces. After applying the masks, the median predicted values extracted from the relevant buffer area of the prevalence location were plotted against observed prevalence values. The quality of the prediction was assessed using Spearman’s rank correlation (r value) between model-predicted and observed values, compared with the line of equality, as well as by plotting the averages of the observed and predicted values against their differences and assessing the proportion of predicted observations within the 95% limits of agreement (q value) [31].

Lastly, we applied the resulting spatial predictions of the best performing models to the population density map in order to estimate the total number of children aged 5–14 years at risk of *S*. *haematobium* and hookworm infection, respectively. For this, we multiplied the population density raster by the predicted prevalence raster in order to obtain the number of individuals at risk within each 30-m cell. We subsequently summed the number of individuals at risk per district.

## Results

### *S*. *haematobium* and hookworm prevalence

**Fig 1:**
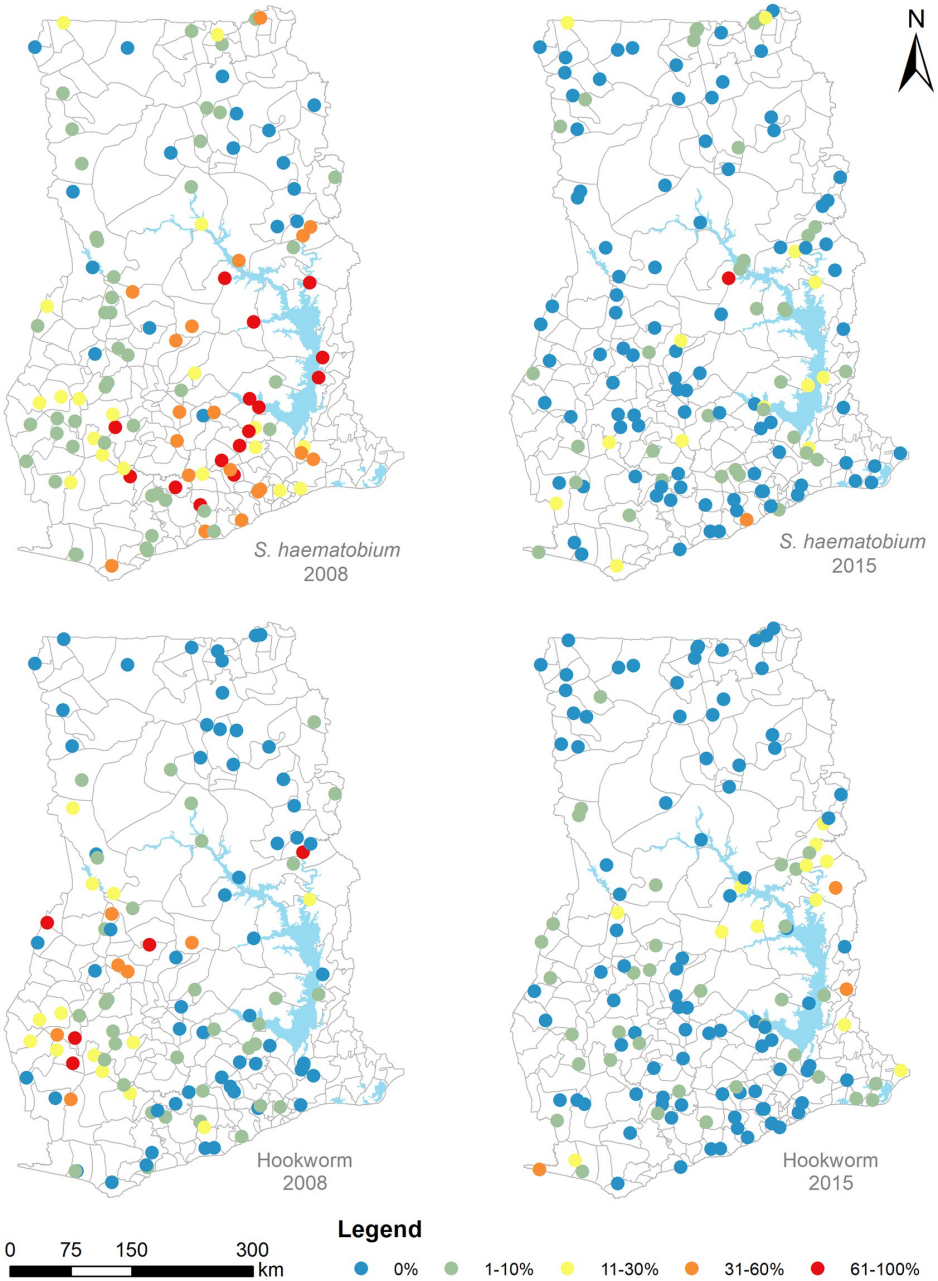
School-based prevalence (%) of *S*. *haematobium* and hookworm in Ghana as estimated from nationally representative surveys conducted by Ghana Health Service in 2008 and 2015. Lakes are shown in blue. Data sources: district boundaries [ArcGIS Hub]; lakes [RCMRD geoportal].

The average school-level *S*. *haematobium* prevalence (with respective standard deviation [SD]) was estimated at 23.8% (SD 27.7%) in 2008 and 3.6% (SD 9.8%) in 2015. Hookworm prevalence also decreased from 8.6% (SD 9.6%) in 2008 to 3.1% (SD 6.9%). Fig 1 shows the spatial distribution of prevalence values.

### Comparison of five buffer distances and two variable extraction methods

Exploratory analyses showed limited variability in predictor variable values across the buffer distances, except the number of pixels within the buffer area that were classified as streams. As expected, the number of stream pixels, and hence, the amount of surface water exposure, increased with increasing buffer distance. There were also no significant differences in predictor value distributions for 2008 and 2015 surveys (S5 Fig).

**Fig 2:**
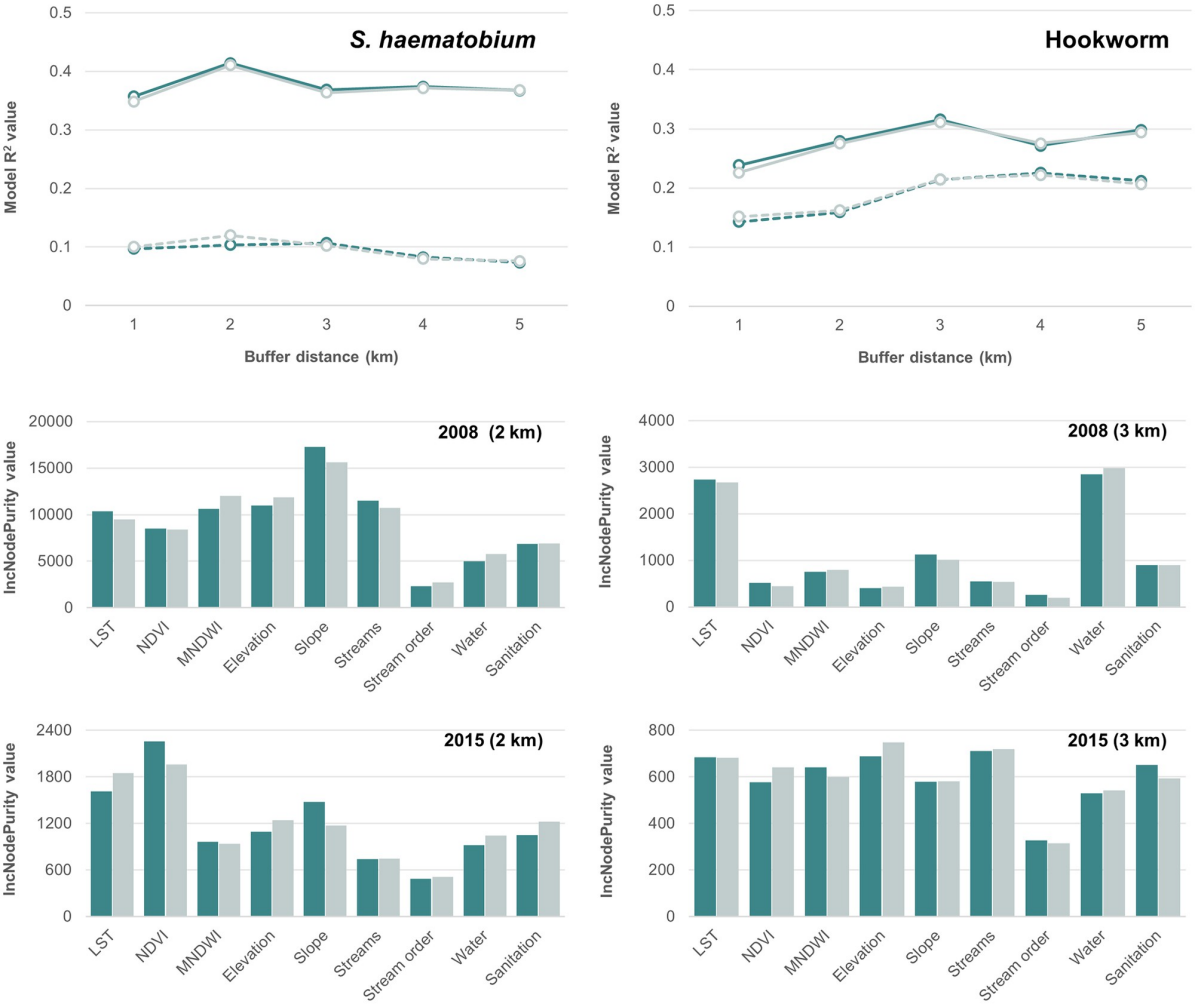
Top row shows R^2^ values (y-axis) across buffer distances (x-axis) for 2008 models (solid line type) and 2015 models (dashed line type) and two masking methods: no mask (dark color) and unpopulated pixels only (light color). Second and third rows show ‘IncNodePurity’ values (y-axis) for each predictor variable (x-axis) for unmasked (dark color) and unpopulated pixels only (light color) extracted from the best performing models.

Results of the random forest models showed better fit for 2008 models as compared to 2015 for both *S*. *haematobium* and hookworm infections (Fig 2). According to model R^2^ values, *S*. *haematobium* models outperformed hookworm models in 2008, whereas hookworm models performed slightly better in 2015. There were no differences in model performance across the two ways of extracting variables (i.e., no mask and unpopulated mask). However, buffer distances were important—peak model performance occurred at the 2 km buffer distance for *S*. *haematobium* and around 3 km for hookworm (Fig 2). According to the unmasked 2 km buffer *S*. *haematobium* model, slope was the predominating explanatory variable in 2008, with the highest variable importance. In the 2015 model, LST and NDVI predominated. According to the 3 km buffer unmasked hookworm model, LST and improved water access were the predominant variables with the highest variable importance. In the 2015 model, most variables had relatively similar variable importance (Fig 2).

### Variable interpretation

**Fig 3:**
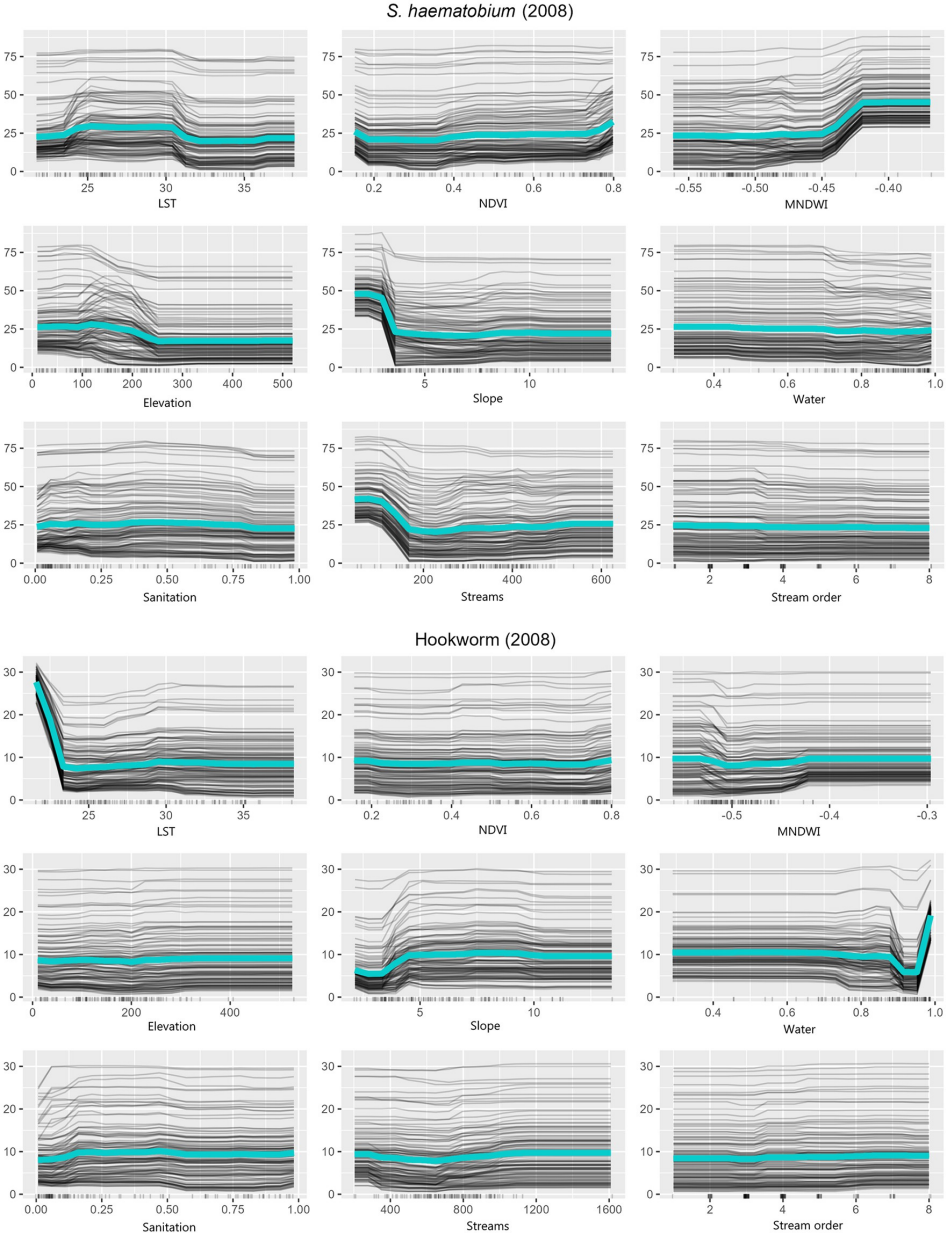
Partial dependence (teal) and individual conditional expectation (gray) plots show the marginal impact of each independent predictor variables (x-axis) on the dependent outcome variable (y-axis) while holding the other variables constant. Only the best performing models were considered: 2 km buffer distance for *S*. *haematobium* and 3 km for hookworm for 2008 survey.

Influence of individual predictor variables on the outcome were interpreted using PD and ICE plots (Fig 3). The teal line represents the PD average, while the gray lines represent how the prediction for parasite prevalence changes as the given predictor variable changes for each instance in the data. This approach helps to visualize how each individual predictor affects the predicted prevalence and makes the prediction process of machine learning models more interpretable [30].

### *S*. *haematobium*

NDVI, water, sanitation, and stream order variables had no visible relationship with *S*. *haematobium* prevalence. LST between 25°C and 30°C was associated with increased *S*. *haematobium* prevalence. MNDWI values of -0.045 and above were also associated with higher prevalence. Increasing elevation and slope decreased prevalence up to 250 m and 3°, respectively, after which there was no relationship. The streams variable was associated with higher prevalence but only up to a total of 170 pixels (30 m^2^ in size) in a 2 km buffer radius (Fig 3).

### Hookworm

NDVI, MNDWI, elevation, sanitation, streams, and stream order variables had no visible relationship with hookworm prevalence. Increasing LST was associated with decreased prevalence up to 17°C, after which there was no relationship. Increasing slope increased prevalence up to approximately 4°. Improved water coverage between 90% and 95% was associated with lower prevalence. However, the highest prevalence was observed at 100% value, likely due to outliers in the dataset (Fig 3).

### Model prediction

**Fig 4:**
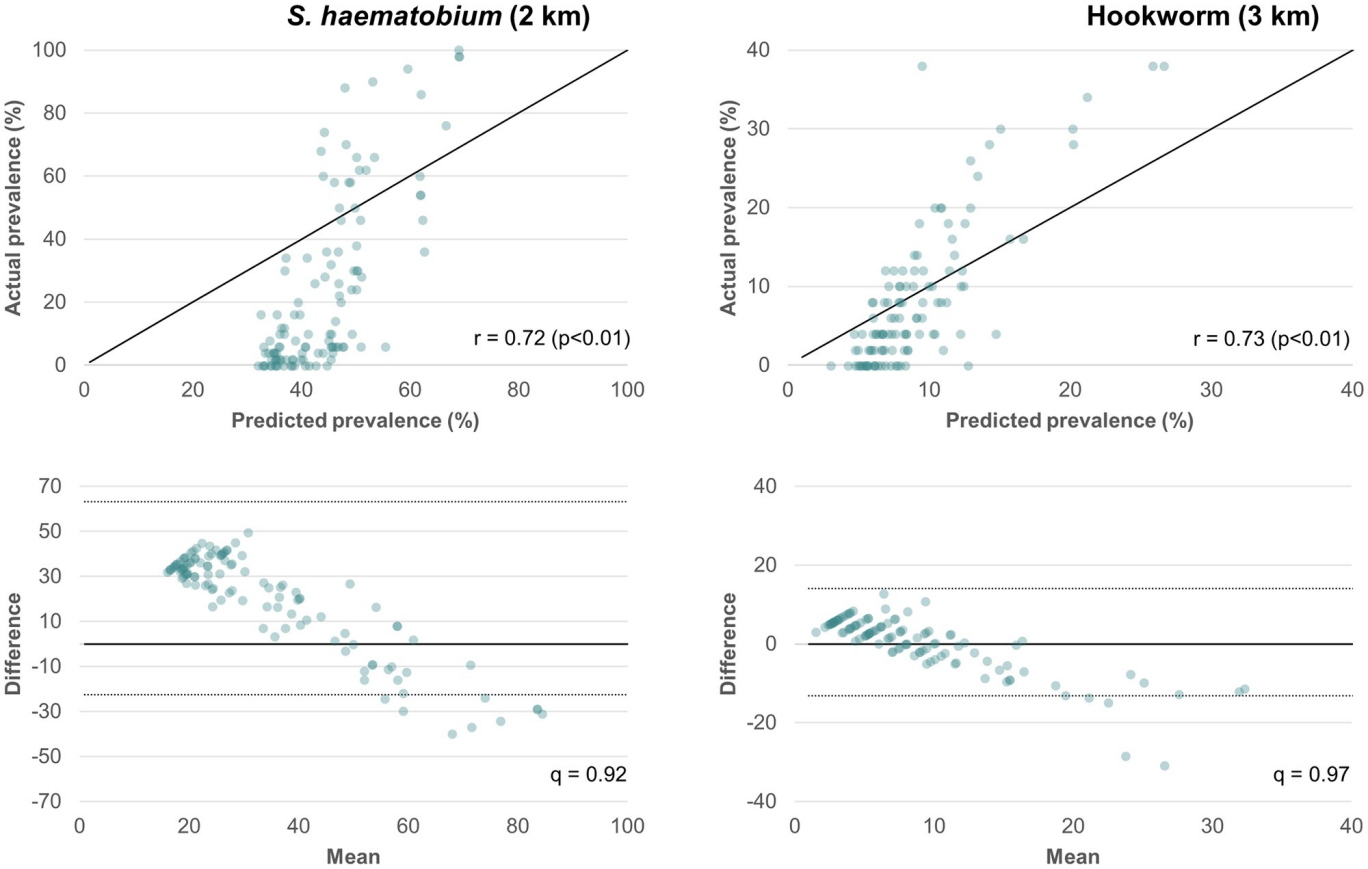
Top row shows a scatter plot of actual (y-axis) versus model predicted (x-axis) prevalence for the unmasked 2008 models as compared to the diagonal line of equality; r is the correlation between observed and predicted values. Bottom row shows a scatter plot comparing the average of observed and predicted prevalence values (x-axis) to the difference between these values (y-axis) with mean difference indicated by a solid line and 95% limits of agreement indicated by dashed lines; q is the proportion of predicted values within the limits of agreement.

**Fig 5:**
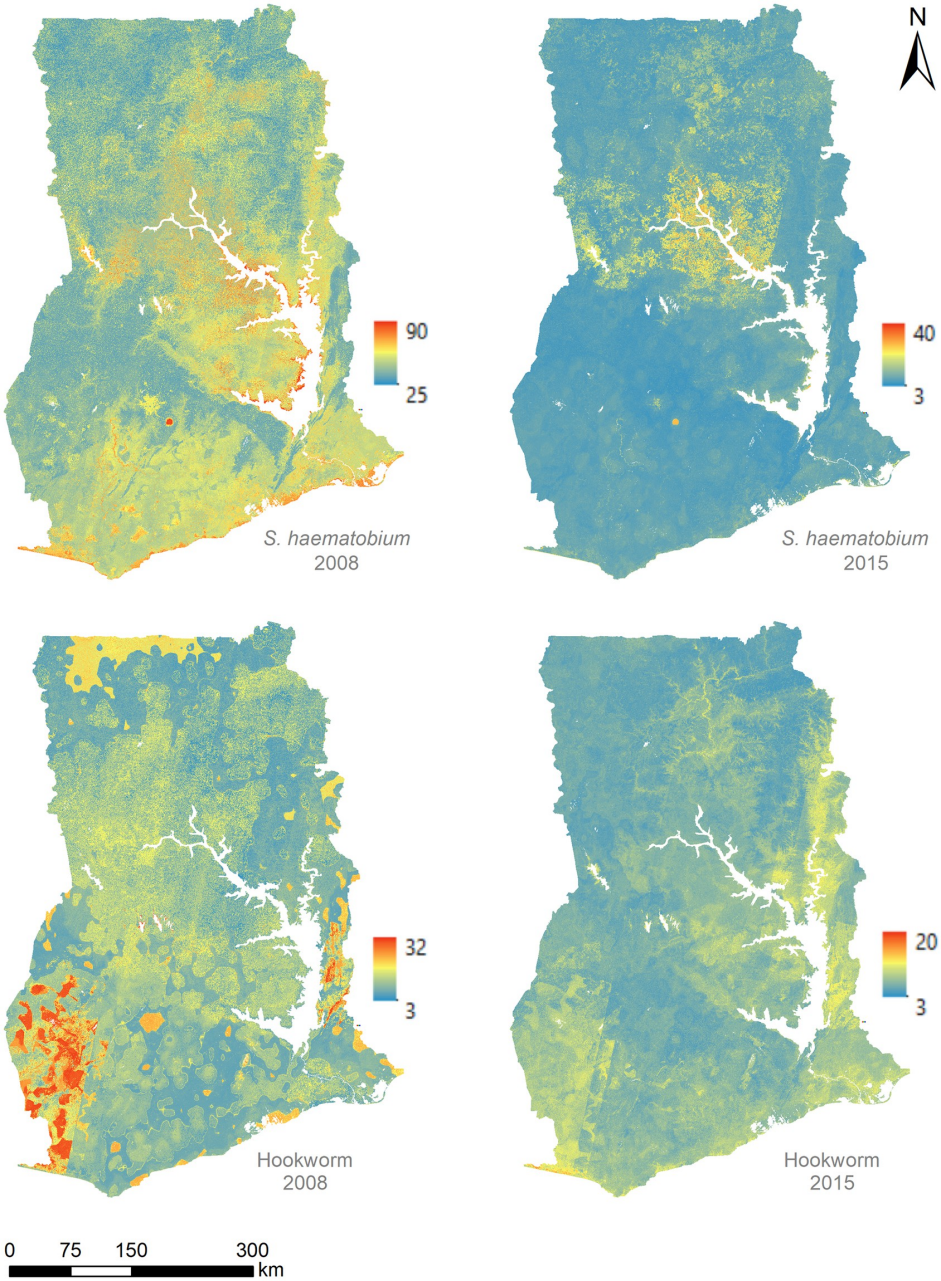
Predicted prevalence values (%) for *S*. *haematobium* (top) and hookworm (bottom) for 2008 and 2015 surveys using unmasked datasets and 2 km and 3 km buffers, respectively. Lakes and areas with significant cloud cover are shown in white. Data sources: Ghana boundary [ArcGIS Hub]; lakes [RCMRD geoportal].

Model prediction was evaluated only on the 2 km buffer radius model for *S*. *haematobium* and 3 km buffer radius model for hookworm. Spearman’s rank correlation values between observed and predicted prevalence were higher for the 2008 survey year than 2015 and very similar for models conducted with unmasked data versus only unpopulated pixels. For *S*. *haematobium*, 2008 and 2015 r values were 0.72 and 0.34 (p<0.01), respectively. For hookworm, the respective values were 0.73 and 0.45 (p<0.01). The mean versus difference plots for unmasked 2008 models illustrated high deviation of predicted prevalence values from the line of equality with actual prevalence values for both *S*. *haematobium* and hookworm (Fig 4). However, a high percentage (>90%) of differences between the observed and predicted values fell within the 95% limits of agreement (Fig 4). Visual assessment of predicted prevalence surfaces (Fig 5) showed highest *S*. *haematobium* prevalence values along the shores of lakes Volta and Bosomtwi and along the coast. Hookworm is most prevalent in the south-western part of Ghana and to the east of Lake Volta. These patterns are well pronounced in the 2008 maps but not in the 2015 maps.

### Population at risk

**Fig 6:**
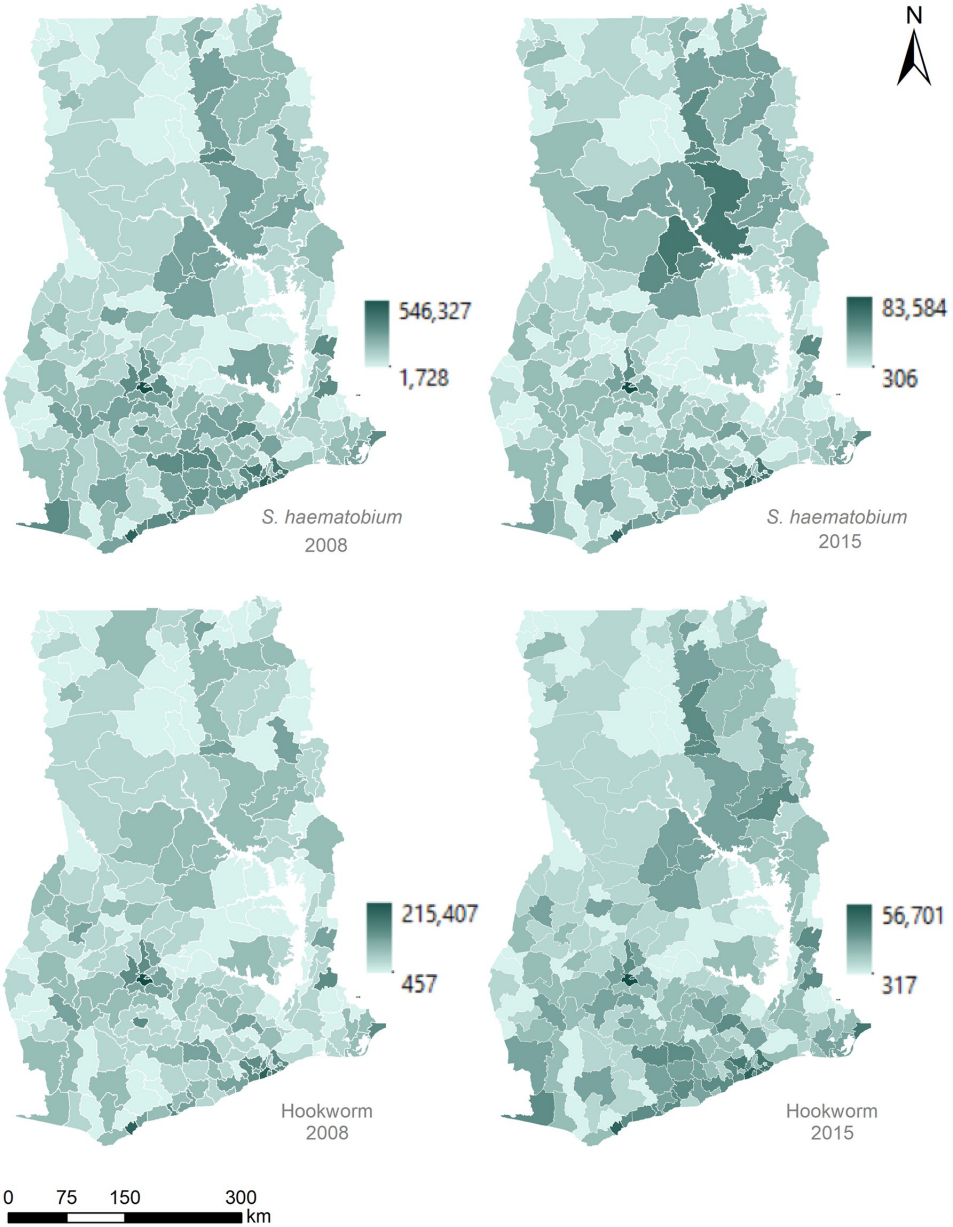
Predicted population (children aged 5–14 years) at risk for *S*. *haematobium* (top) and hookworm (bottom) per district (n = 216) generated using the four models using 2020 population estimates. Lakes are shown in white. Data sources: district boundaries [ArcGIS Hub]; lakes [RCMRD geoportal].

Population at risk in the age group 5–14 years was estimated using the population density data for 2020 and aggregated at the district level (Fig 6). For *S*. *haematobium* infections, the magnitude of the population at risk was significantly reduced from 5,016,461 in 2008 to 744,652 in 2015 (a decline of 85.2%). For hookworm infections, the reduction was less drastic from 1,241,057 in 2008 to 631,089 in 2015 (a decline of 49.1%) (S6 Fig). Spatially, the districts with relatively higher populations at risk persisted from 2008 and 2015 for both helminth species (Fig 6). Likewise, there was a correlation between areas at higher risk of *S*. *haematobium* and hookworm, indicating co-infections [15].

## Discussion

We analyzed publicly available environmental data in combination with topographic and WASH variables to assess their performance in predicting *S*. *haematobium* and hookworm prevalence before (2008) and after (2015) the launch of large-scale preventive chemotherapy programs in Ghana. We examined two methods of environmental data extraction and a variable buffer radius for environmental variable aggregation around point-prevalence locations. We used interpretable machine learning methods to improve the understanding of which context variables contributed most to the model predictions. We also demonstrated the use of a new population density dataset in estimating the population at risk based on the model-predicted prevalence.

We found that after several rounds of preventive chemotherapy with high reported coverage, the average school-level prevalence of *S*. *haematobium* and hookworm declined significantly from 23.8% to 3.6% and from 8.6% to 3.1%, respectively. The stark prevalence declines are similar to those observed in other sub-Saharan African countries [9,32]. As expected, environmental model performance declined for both helminth infections. At risk areas largely maintained their geographic clusters from the 2008 to 2015 models, with the same districts displaying relatively higher treatment needs over time. A recent modeling study recommended a prevalence threshold of 5–11% among school-age children to indicate control of *S*. *haematobium* in a geographic location [33]. Another study suggested that school-based preventive chemotherapy is cost-effective for schistosomiasis at a prevalence threshold of 5% and for soil-transmitted helminthiasis at a prevalence threshold of 20% [34]. In the 2015 survey, 17% of the surveyed schools exceeded the 5% prevalence threshold for *S*. *haematobium* and 4% exceeded the 20% prevalence threshold for hookworm.

Generally, higher values of MNDWI correlated with higher *S*. *haematobium* risk, as expected. LST between 25°C and 30°C was associated with increased *S*. *haematobium* risk, consistently with the documented favorable temperature range for snail and cercariae survival [35,36]. Higher elevation and steeper slope (indicating higher stream flow velocity) decreased risk, which is also in line with the extant literature. However, all elevations observed in the analysis were far below the 2,000 m threshold for *S*. *haematobium* transmission [37]. In the case of hookworm, increasing LST was generally associated with decreased risk, as has been found in other studies [38] and increasing slope was associated with increased risk of infection. Water and sanitation failed to predict either infection, potentially due to highly generalized coarse spatial resolution (5 km) of the DHS dataset and the fact that data of 2015 are likely not representative of conditions in 2008.

We found that buffer distance is worthy of consideration in modeling. The 2 km buffer radius resulted in best model performance for *S*. *haematobium* and the 3 km radius performed best in the hookworm models. The methods of data extraction, or masks, however, did not make a difference in this analysis, consistent with our prior work at a smaller sub-national spatial extent. The preceding study demonstrated that extracting environmental data only from known water bodies or water contact locations results in improved model performance, as compared to unmasked or unpopulated pixels, which had similar model performance [23]. We conclude that the use of masking is only relevant if specific water contact locations are known. We do recommend the use of the variable buffer radius when using RS datasets with fine spatial resolution. With coarse spatial resolution datasets, the use of the buffer radius is less relevant.

Overall, the models had relatively low predictive power and predicted prevalence values deviated substantially from the observed values, indicating over prediction in the low-prevalence range and under prediction in the high-prevalence range. This has implications for the success of these modeling approaches in the era of widespread preventive chemotherapy. The study was limited by Landsat 8 data availability—data were not available for 2008; hence, 2015 data were used in both models after validation against another free dataset that confirmed that there were no significant climatic changes during this period. However, some regions did experience more significant change in their climate than others, which could have affected the analysis.

Another significant challenge to our analysis was posed by cloud cover in RS data acquisition. For countries like Ghana, cloud-free images are typically only available during the dry season. This limitation especially affects variables such as LST, which unlike vegetation or water indices, experiences shorter-term changes, making a single image less reliable. This is especially difficult in the case of NTD modeling, where the timing of prevalence data acquisition, the most likely timing of exposure, and the links to environmental conditions must be considered. In our analysis, LST represented by images extracted during November and December represents the higher range of annual temperatures, close to the maximum, which is most relevant in our case. In other use-cases, this approach may not be relevant and possible implications on the validity of the conclusions should be carefully examined.

Despite these limitations, our study contributed to a growing body of modeling studies for spatiotemporal risk profiling of *S*. *haematobium* and hookworm transmission. First, we justified the use of a variable buffer radius for extracting environmental variables to link with point-prevalence data. In Ghana, the radius of 2–3 km resulted in the best model performance. Second, our analysis further supported the use of MNDWI as the preferred water index in *S*. *haematobium* modeling, corroborating our previous findings [23]. Third, we improved on the black-box random forest modeling approach by incorporating visual interpretation of the results. This relatively simple and interpretable approach could be utilized by public health agencies for risk profiling of environmental diseases. We do advise to use the approach with caution, and to be mindful of the R^2^ values and model prediction error. We provide the methodology to do so (Fig 4). The use of RS data in the modeling approach is likely more relevant for diseases that are affected by changes in the environment (e.g., deforestation, drought, flooding) but for which no concurrent large-scale pharmaceutical interventions are taking place. Alternatively, RS data can be used to monitor the change in environmental conditions (e.g., changes in waterbodies in the case of schistosomiasis risk), rather than disease prevalence, as an endpoint.

In conclusion, most current modeling approaches for NTD risk profiling continue to rely on environmental variables. However, with declining prevalence in the face of large-scale control programs emphasizing a pharmaceutical intervention (i.e., annual deworming), associations with the environment weaken and predictive power of the models also declines. Despite falling prevalence, hotspots of infections persist, as treatment programs may be incomplete (e.g., not reaching out-of-school children or adults) and water-related risk factors are not mitigated with persisting high rates of reinfection. In light of these trends, it is timely to develop new cost-effective passive surveillance methods (e.g., improving diagnosis and reporting at primary care level) for NTDs. Further, we recommend re-evaluating the use of RS data for modeling of environmental diseases as endpoints, for which pharmaceutical interventions are in place.

## Supporting information

S1 Data 40 anonymized datasets used in the analysis. (XLSX)

S1 Fig Map of Ghana showing regional [10] and district (216) boundaries. Data sources: region boundaries [ArcGIS Hub]; district boundaries [ArcGIS Hub].
(TIFF)

S2 Fig Comparison of monthly (x-axis) climate parameters (y-axis) in the 10 regions of Ghana for 2008 (dark color) and 2015 (light color). Bar plot represents cumulative rainfall (mm) displayed on the left y-axis. Line plot represents average normalized difference vegetation index (NDVI) displayed on the right y-axis. Data source [WFP dataviz].
(TIFF)

S3 Fig Data processing and analysis steps. (TIF)

S4 Fig Collection of Landsat images making up the spatial extent of Ghana that were screened for quality. In the map, shading of the tiles does not have meaning. In the table, dates in white have <10% of pixels affected by clouds. Dates in gray have 10% or more of the pixels affected by clouds. Dates outlined in bold were selected for analysis and mosaicked. Data sources: Ghana boundary [ArcGIS Hub].
(TIFF)

S5 Fig Boxplots comparing the distribution of aggregated predictor values in the unmasked dataset across buffer distances for the locations of 2008 survey (dark color) and 2015 survey (light color). (TIFF)

S6 Fig Predicted number of children aged 5–14 years per district (n = 216) at risk of *S*. *haematobium* (top) and hookworm (bottom) infection according to the 2008 model (light color) and 2015 model (dark color). Both estimates are as applied to 2020 population data.
(TIFF)
