# Supplementary figures and images for: How do disease control measures impact spatial predictions of schistosomiasis and hookworm? The example of predicting school-based prevalence before and after preventive chemotherapy in Ghana
Source: PLoS Negl Trop Dis. 2023 Jun 16;17(6):e0011424. doi: 10.1371/journal.pntd.0011424 (PMC10310024; doi:10.1371/journal.pntd.0011424)

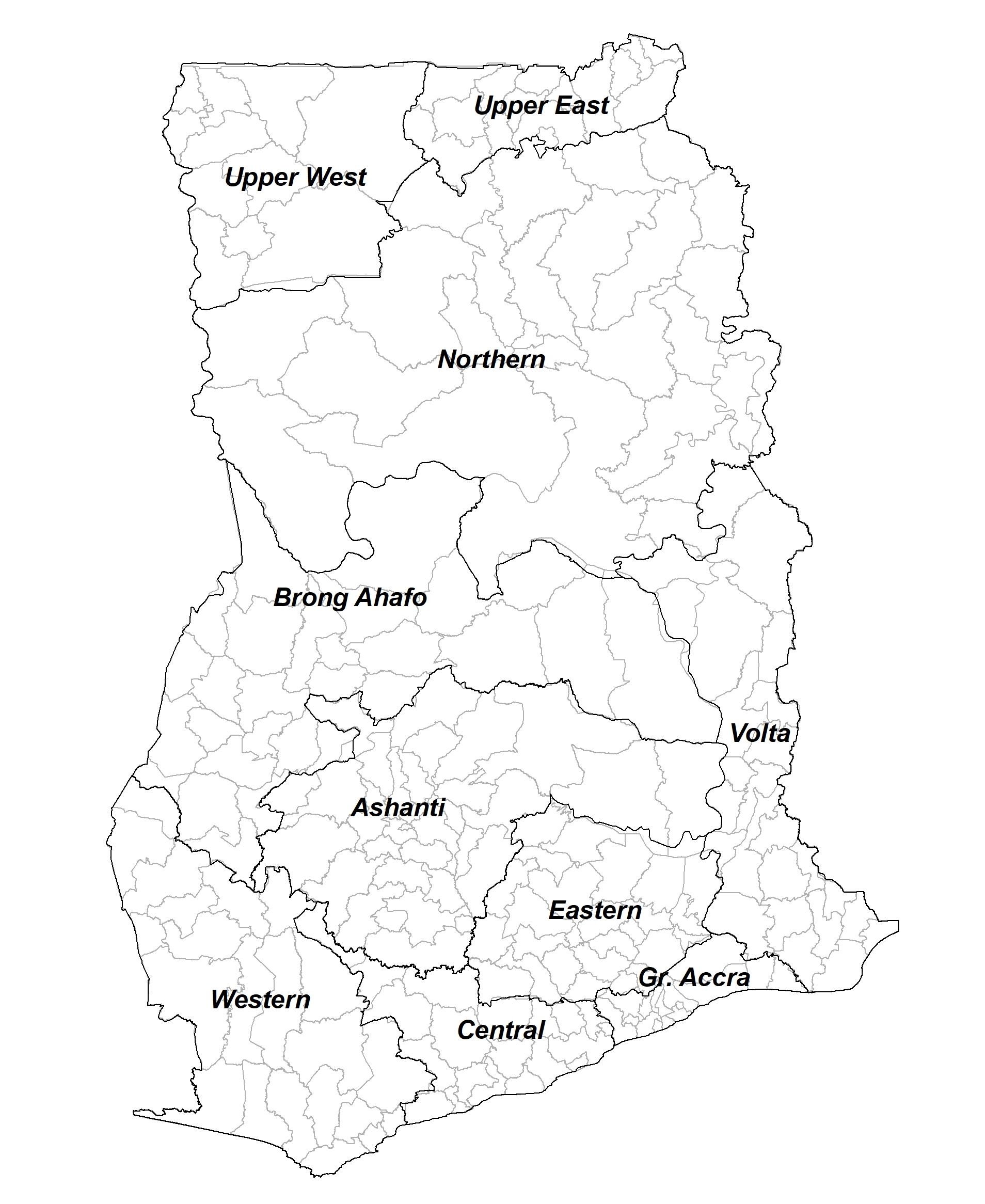

Supplement: S1 Fig — Data sources: region boundaries [ArcGIS Hub]; district boundaries [ArcGIS Hub]. (TIFF) [file pntd.0011424.s002.tiff]

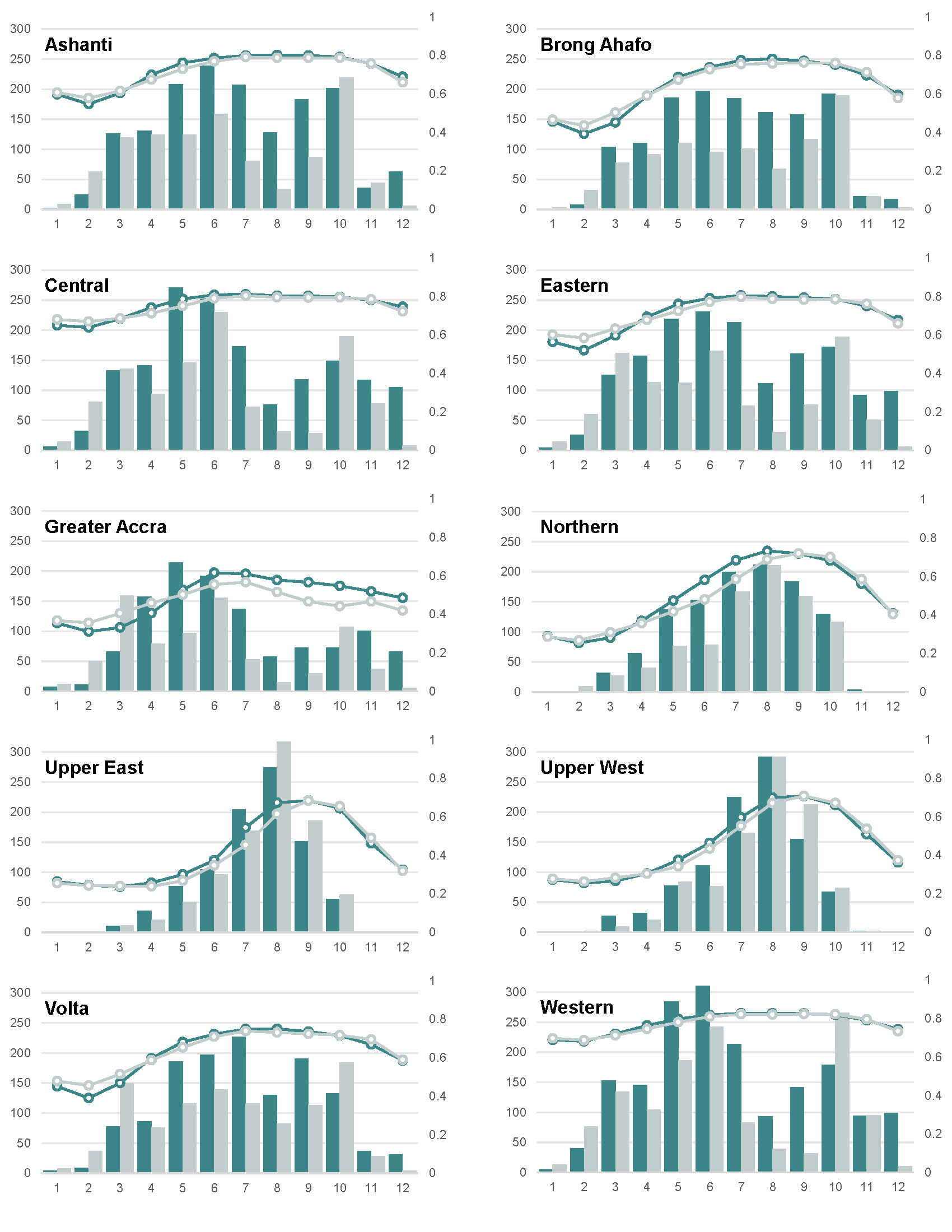

Supplement: S2 Fig — Bar plot represents cumulative rainfall (mm) displayed on the left y-axis. Line plot represents average normalized difference vegetation index (NDVI) displayed on the right y-axis. Data source [WFP dataviz]. (TIFF) [file pntd.0011424.s003.tiff]

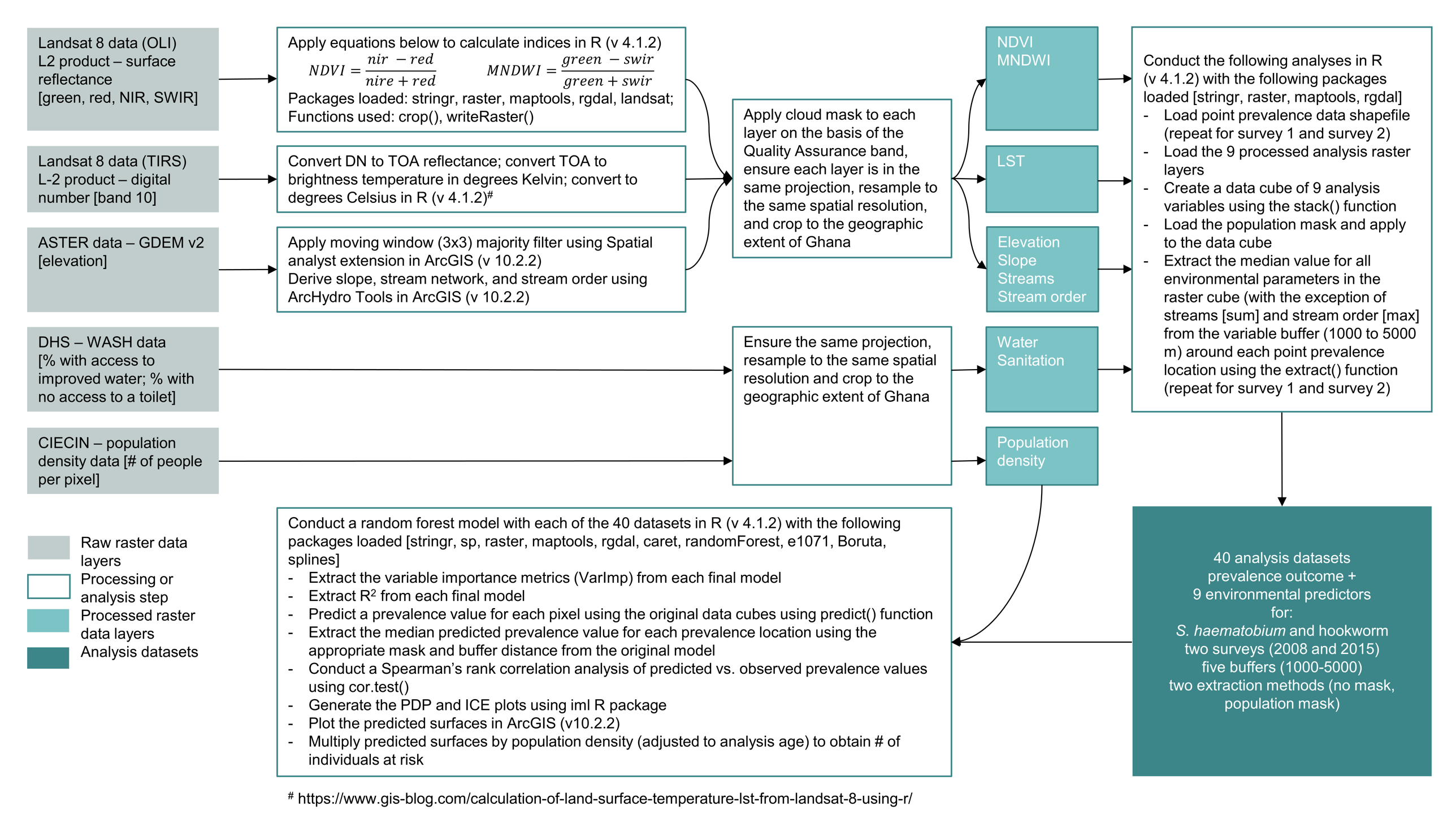

Supplement: S3 Fig — (TIF) [file pntd.0011424.s004.tif]

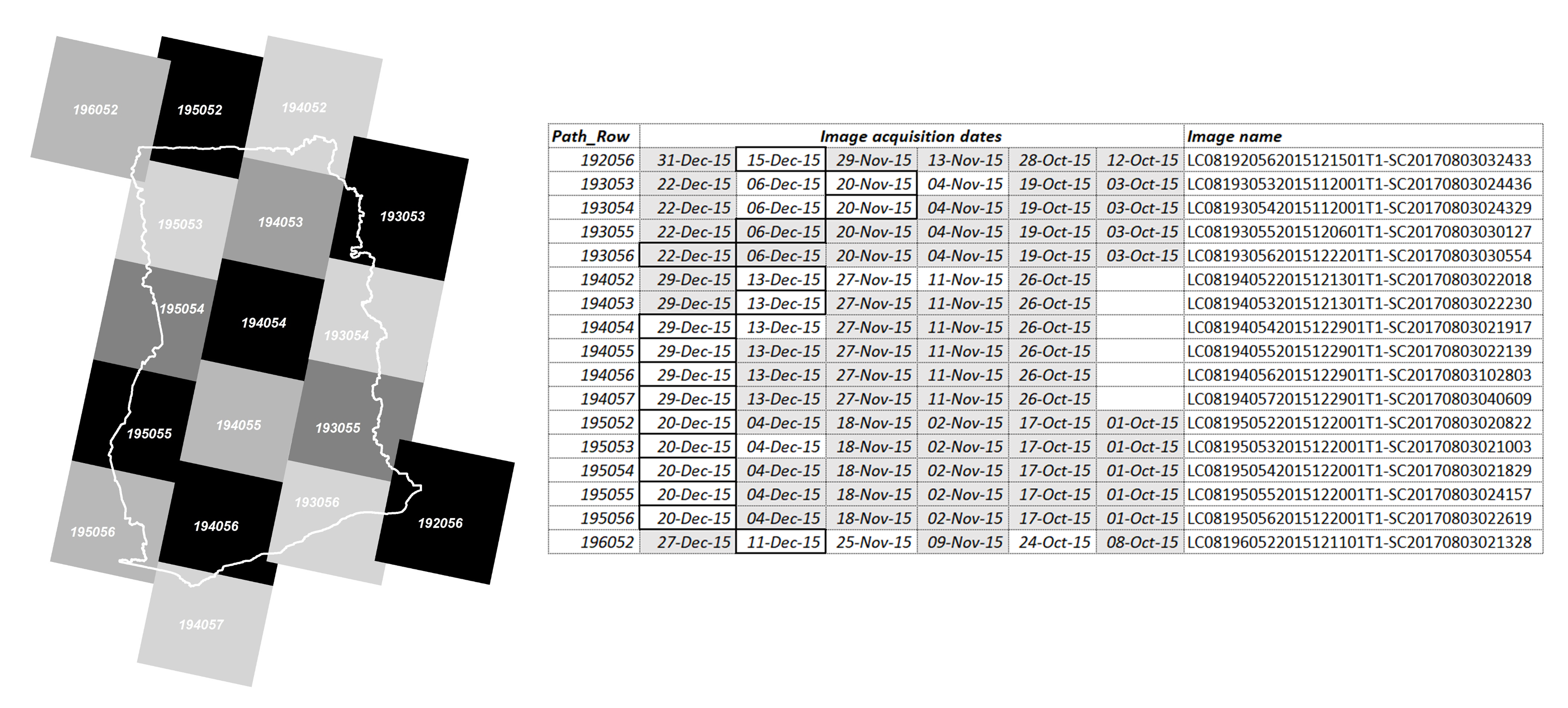

Supplement: S4 Fig — In the map, shading of the tiles does not have meaning. In the table, dates in white have <10% of pixels affected by clouds. Dates in gray have 10% or more of the pixels affected by clouds. Dates outlined in bold were selected for analysis and mosaicked. Data sources: Ghana boundary [ArcGIS Hub]. (TIFF) [file pntd.0011424.s005.tiff]

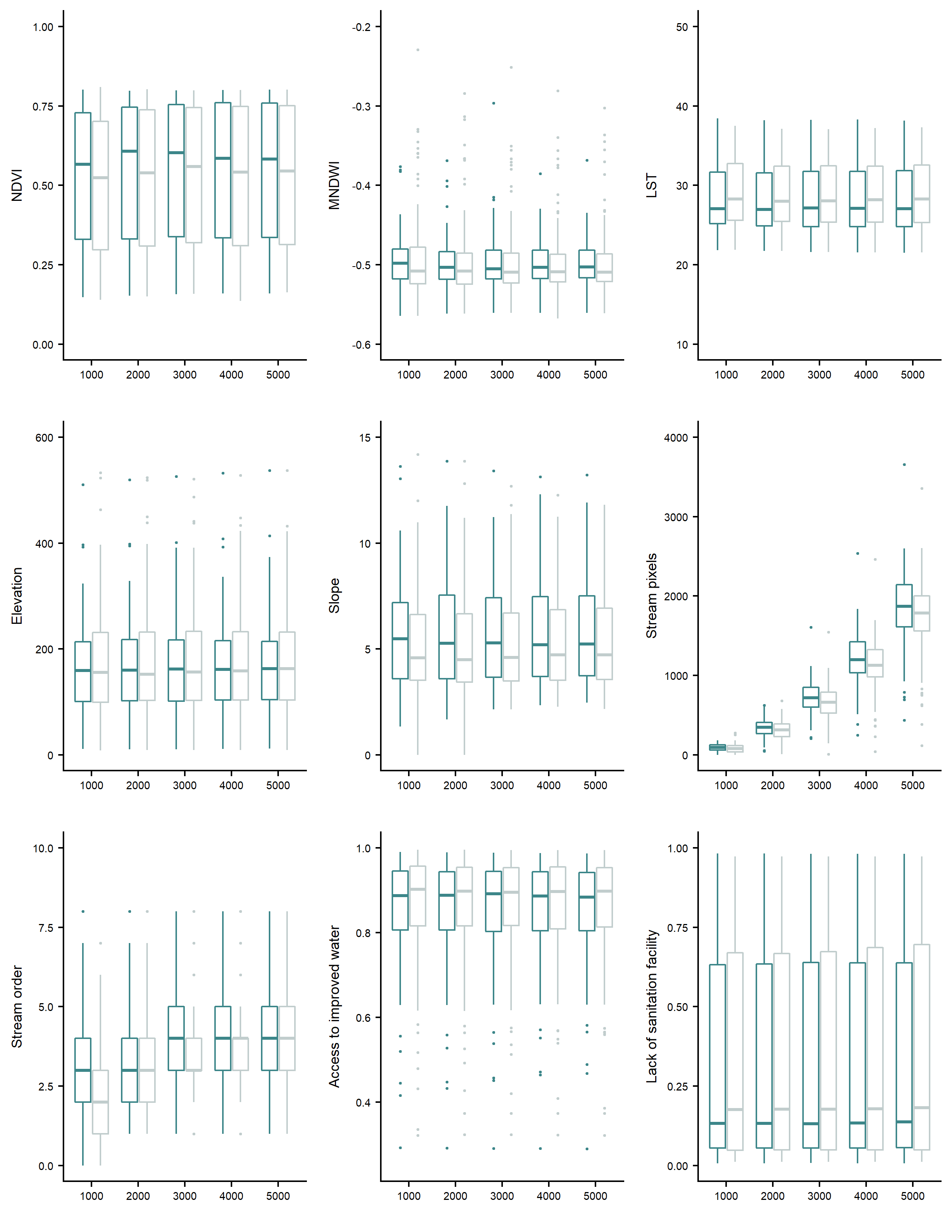

Supplement: S5 Fig — (TIFF) [file pntd.0011424.s006.tiff]
